# F@ce: a team-based, person-centred intervention for rehabilitation after stroke supported by information and communication technology – a feasibility study

**DOI:** 10.1186/s12883-020-01968-x

**Published:** 2020-10-23

**Authors:** Susanne Guidetti, Martha Gustavsson, Kerstin Tham, Magnus Andersson, Uno Fors, Charlotte Ytterberg

**Affiliations:** 1 Division of Occupational Therapy, Department of Neurobiology, Care Sciences and Society, Karolinska Institutet, Huddinge, Sweden; 2 Faculty of Health and Society, Malmö University, Malmö, Sweden; 3 Department of Clinical Neuroscience, Karolinska Institutet, Solna, Sweden; 4 Department of Computer and Systems Sciences (DSV), Stockholm University, Stockholm, Sweden; 5 Division of Physiotherapy, Department of Neurobiology, Care Sciences and Society, Karolinska Institutet, Huddinge, Sweden; 6 Function Area Occupational Therapy & Physiotherapy, Karolinska University Hospital, Stockholm, Sweden

**Keywords:** ADL, Disability, eHealth, Mobile phone, Occupational therapy, Participation in everyday life, Physiotherapy, Tablet, Tele rehabilitation, Telehealth

## Abstract

**Background:**

Globally, there is a growing use of Information and Communication Technology (ICT), including mobile phones, tablets and computers, which are being integrated into people’s daily activities. An ICT-based intervention called F@ce was developed in order to provide a structure for the process in stroke rehabilitation and facilitate change by integrating a global problem-solving strategy using SMS alerts. The aim of the study was to evaluate the feasibility of i) F@ce within in-patient and primary care rehabilitation after stroke, ii) the study design and outcome measures used, and iii) the fidelity, adherence and acceptability of the intervention.

**Methods:**

Three teams comprising occupational therapists and physiotherapists who work in neurological rehabilitation participated in a preparatory workshop on F@ce and then enrolled 10 persons with stroke to participate in the intervention. Goals were set using the Canadian Occupational Performance Measure (COPM) and the participants with stroke rated their performance and satisfaction with the activities associated with the three goals every day for 8 weeks. Data were collected at inclusion, at four and 8 weeks, using the COPM, Stroke Impact Scale, Frenchay Activities Index, Life Satisfaction Checklist, Self-Efficacy Scale, Hospital Anxiety and Depression Scale, Fatigue Severity Scale, follow-up survey, daily ratings on the web platform and logbooks.

**Results:**

All of the participants showed increased scores in the primary outcome (COPM) and a clinically meaningful improvement of ≥2 points was found in four participants regarding performance and in six participants regarding satisfaction. Overall fidelity to the components of F@ce was good. The response rates to the F@ce web platform were 44–100% (mean 78%). All of the participants stated that F@ce had supported their rehabilitation.

**Conclusion:**

The results indicate that the most beneficial part of F@ce was the person-centred, goal-setting process and SMS alerts. All participants were satisfied with F@ce and highlighted the benefits of receiving daily alerts about their goals. This encouraged them to be more active. The only downside mentioned was that they felt under an obligation to practice, although this was described as “a positive obligation”.

**Supplementary information:**

**Supplementary information** accompanies this paper at 10.1186/s12883-020-01968-x.

## Background

Digitalisation in society, as well as in health care and rehabilitation, has increased rapidly in recent years [1, 2]. In line with this development, the Swedish government has created a vision of becoming a global leader in digital health solutions by 2025 [2]. Digitalisation can be a valuable tool for increased participation in society for people with disabilities as after stroke [3, 4]. There are a range of concepts and definitions that address different aspects of digitalisation in health care such as e-health, tele rehabilitation and health informatics. In this study, the term *Information and Communication Technology* (ICT) is used, including all technologies that are used interactively for communication and transfer of information, such as mobile phones, tablets and computers, as well as the applications and software of such devices [5].

The ability to manage activities in daily living (ADL) and participate in everyday life, including work, leisure and social activities, is often restricted after a stroke [6–9]. Thus, the everyday life after stroke has been described as chaotic and receiving rehabilitation to manage ADL is often a priority [8, 10]. The development of more user-friendly ICT solutions has created opportunities to provide ICT-supported rehabilitation services that could reduce some of the unmet needs of rehabilitation that are reported by people who have had stroke [9]. Although the evidence concerning the effectiveness of ICT is inconclusive [3, 4], a recent review has shown that interventions using ICT have beneficial effects on motor, higher cortical and mood disorders [4]. It has also been shown that ICT used as an alternative to face-to-face interventions could improve participation in daily life after stroke [3].

ICT could be utilised in rehabilitation after stroke to monitor rehabilitation progress and interact at a distance [3, 4]. The use of a mobile phone or computer has been shown to promote participation in everyday life and create a sense of security [11, 12]. Furthermore, the use of ICT-based interventions could reduce the number of home visits, thereby saving time and travel costs, particularly in rural areas [12, 13]. ICT solutions have also shown to enable person-centred care [14, 15] and facilitate communication and feedback from healthcare professionals [3, 16]. A concern among people with stroke is their potentially limited ability to manage different ICT devices. Earlier research has found that people could encounter a range of difficulties [11, 17, 18] but that people with acquired brain injury such as stroke could benefit from using ICT in their daily lives [11, 19]. Moreover, ICT could be successfully introduced and used within rehabilitation after acquired brain injury, regardless of age or previous use [20]. However, support is often needed, particularly when using a new device or when something unexpected happens [11].

A client-centred ADL intervention (CADL) was developed with the aim of enabling agency in activities and participation in everyday life among persons with stroke [21, 22]. The CADL was based on phenomenology with the lived experiences of the person as a point of departure for the intervention [23]. The client-centred approach included building a therapeutic relationship and ensuring that the person was actively involved in the goal setting and planning of the rehabilitation [24–26]. The CADL was delivered by occupational therapists and evaluated in a randomized controlled trial (RCT) [21, 22] along with qualitative studies [27–29]. The results of the RCT [21, 22] were inconclusive but the qualitative studies emphasized that sharing [28] and transparency [29] between therapists and the patients were benefits of using a client-centred approach. It was also shown that the CADL appeared to enhance the involvement of patients in goal setting and individualisation of the rehabilitation. In the present study, the CADL was further developed by following the Medical Research Council (MRC) guidelines for the development of complex interventions [30].

The results of the CADL study is a part of the evidence base in the development of the new intervention called F@ce that is presented in this study. One conclusion from the CADL evaluations was that all members of a stroke rehabilitation team should use the intervention. This is also recommended in the Swedish national guidelines for stroke care [31] and in this new intervention F@ce, the multidisciplinary teams were included. In line with the new multidisciplinary approach, the term *client-centred* was replaced with *person-centred*. The terms *client-centred* and *person-centred* are based on the same underlying theories as described by Rogers [32]. The person-centred approach views the person as having the potential to change and the therapist as being a facilitator in this process [32, 33].

The potential benefits and obstacles for using ICT within a person-centred rehabilitation intervention for people after stroke remain largely unexplored. Although healthcare professionals and persons with stroke have reported high levels of acceptance and satisfaction when using ICT interventions in stroke care, few studies have explored the outcome of such interventions [3, 34]. Thus, to meet the vision of the Swedish government [2], further research on the development and use of ICT within rehabilitation is needed.

Our assumption was that ICT could be used as a tool for reinforcing person-centred rehabilitation through increased sharing [28] and transparency [29]. According to the MRC guidelines, an important stage in the development of new interventions is conducting a feasibility study before testing on a larger scale [30]. Thus, this study had the following aim: *to evaluate the feasibility of i) F@ce within in-patient and primary care rehabilitation after stroke, ii) the study design and outcome measures used, and iii) the fidelity, adherence and acceptability of the intervention.*

## Method

### Study design

A single group design was used to evaluate the feasibility of F@ce, a person-centred, team-based intervention for rehabilitation after stroke supported by ICT. The CONSORT 2010 statement for randomised pilot and feasibility trials [35] was used as a structure (available as Supplementary Material).

### Recruitment of participants with stroke

The six team members were responsible for identifying and recruiting 4–5 participants with stroke from each unit in September 2017. The inclusion criteria for the person with stroke were 1) referred to one of the participating units, 2) able to participate in an eight-week intervention, and 3) able to communicate in Swedish. Since a stroke often leads to long-term consequences on the person’s ability to return to daily life [36, 37], the team members suggested that it would be beneficial to evaluate F@ce also after the initial rehabilitation period i.e. to recruit participants regardless of time since stroke.

Professionals at the in-patient unit only recruited participants with stroke who, following discharge, could continue the F@ce intervention at one of the two participating primary care rehabilitation units. One of the participating team members notified persons with stroke who met the inclusion criteria about the study and those persons who agreed to participate signed a written consent form. The recruitment process was documented in logbooks kept by the team members and in field notes taken by the second author. In this feasibility study, a prospective sample size was not calculated.

### Setting and recruitment of staff

Convenience sampling was used for recruitment of professionals to carry out F@ce. In total, six professionals, members of rehabilitation teams at one urban hospital based inpatient rehabilitation unit and two corresponding primary care rehabilitation units were recruited. Other professionals were also part of the teams, including speech and language therapists, medical social workers and dieticians. However, these professionals often worked in several different teams and were only involved as consultants when needed. The teams at the in-patient units also included physicians and nurses who were responsible for medical care.

### The F@ce

#### The training of team members

**Fig. 1:**
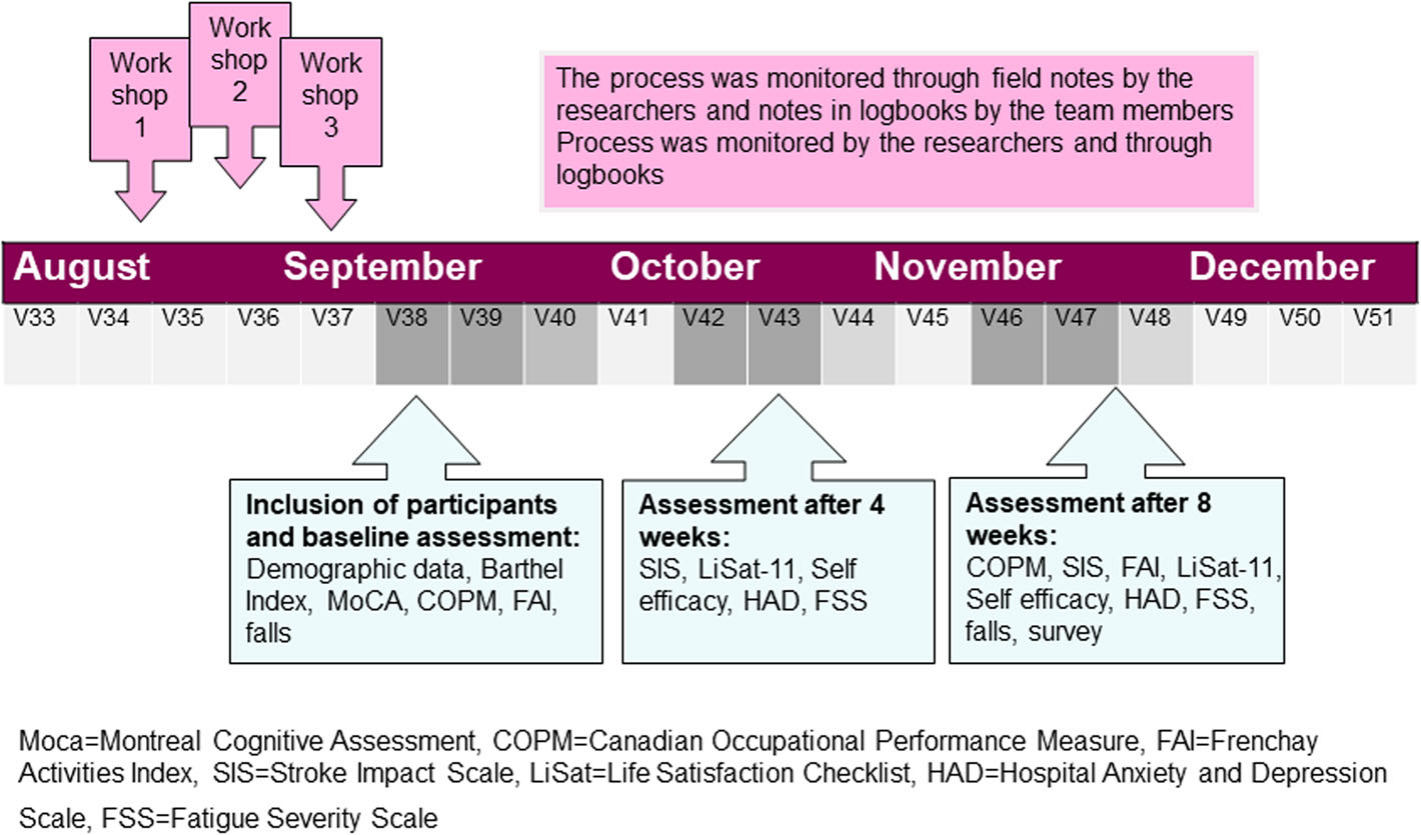
Timeline of the study. The process of the study including the data collection timeline and the instrument used

The team members participated in three workshops, 2 h each week for 3 weeks. The timeline is presented in Fig. 1. The aim of the workshops was to deepen the knowledge of person-centredness and participation and become familiar with the components of the F@ce intervention. The workshops contained short presentations on the theories, concepts and research underlying F@ce, as well as practical exercises of the goal setting and ICT used in the intervention.

#### The intervention

The F@ce intervention was an eight-week intervention that aimed to increase perceived participation in everyday life and self-efficacy, and to reduce the impact of stroke. The F@ce intervention was based on three basic principles from the CADL: applying a person-centred approach, enabling sharing throughout the rehabilitation process and using a transparent goal-setting process (see Supplementary material, Fig. 3).

The first basic principle of the F@ce intervention was the person-centred approach. The team members focused on building a relationship with the person with stroke [33, 38] by listening to the person’s narrative [33] and unique life experiences [23, 39] in order to understand their previous habits, roles and how they performed daily activities [25].

The second basic principle of the F@ce intervention was sharing throughout the rehabilitation process [28, 34]. For example, the team members and the person with stroke watched a video recording of an activity performed by the person with stroke in order to gain a shared overview of the person’s abilities.

The third basic principle of the F@ce intervention was using a transparent goal-setting process [38]. The Canadian Occupational Performance Measure (COPM) [40] was used to set goals and evaluate after 8 weeks when the intervention had ended. Three goals, based on activities that the person with stroke needed and/or wanted to do, as well as strategies for supporting the performance of the activities, were formulated in collaboration with the team.

### ICT used within the project

#### Website

The research team developed a website including contact information of the participating researchers and reference literature for the intervention. The aims and content of each workshop were also available on the website to enable the team members to prepare and reflect before and after each workshop.

#### Stroke rehabilitation platform

A web-based platform, password protected, for the F@ce intervention was developed with three interfaces: 1) an administrator view used by the researchers in order to monitor the F@ce intervention process, 2) a team view in which team members registered the results of the COPM and monitored their patients’ daily ratings, and 3) a view for the participants with stroke in which daily alerts regarding their goals and strategies were set by the researcher and sent automatically as an SMS from a server at Stockholm University to the participant’s mobile phone or tablet each morning. This was followed by a short survey each afternoon that asked the following question: “How well did you manage to work on your goals today?” on a scale from 1 (did not perform any activity) - 5 (performed the activity very well). A low rating (1, 2) was marked in red in the system, a medium rating (3) was marked in yellow and a high rating (4, 5) was marked in green. The goals were transparent since they were visible and available to the participants on their mobile phones.

#### Database

An online, password protected database was developed for data collection used by the researcher. The results were then stored on a secure server.

### Data collection

The timeline for the data collection is presented in Fig. 1. Demographic data on age, gender, months post stroke, employment before stroke and living situation were collected from the persons with stroke by the second author within the first week of inclusion. The persons with stroke were also asked to choose from three response categories in relation to their current use of ICT: Basic *(use their mobile phones to make calls and receive text messages only)*, Moderate *(use a smartphone and/or a tablet for searching on the internet)* and Advanced *(use a smartphone and/or a tablet and/or a computer for advanced activities)*.

Stroke severity was assessed using the Barthel Index [41]and scores were categorized as severe (< 15), moderate (15–49) or mild (50–100). Cognitive function was assessed using the Montreal Cognitive Assessment (MoCA) [42] with a score range of 0–30 and a score of < 26 indicating cognitive impairment. In total these baseline assessments took about 20–40 min to perform.

### Primary outcome measures

The COPM [40] was used at inclusion and after the intervention to assess performance and satisfaction with self-care, productivity and leisure from the perspective of persons with stroke. The participants were first asked to rate their performance in the three chosen activities on a scale ranging from 1 (extremely poor/could not manage) to 10 (extremely well) and, secondly, to rate their satisfaction with their performance using the same scale. Weighted scores of performance and satisfaction of the chosen activities were summarized separately to create two total scores. A change of two points was seen as a clinically meaningful change [40].

The Stroke Impact Scale (SIS) 3.0 [43] was used at 4 weeks after inclusion to assess the perceived impact of stroke over the last 1–4 weeks. The SIS 3.0 has eight domains: strength, memory and thinking, emotions, communication, ADL/IADL, mobility, hand function, and participation. The scores range from 0 to 100 and the higher the score the less the impact of stroke. In addition, perceived recovery after stroke is rated on a visual analogue scale ranging from 0 (no recovery) to 100 (full recovery). An increased score of ≥15 points has been defined as constituting a clinically meaningful change [44].

The Frenchay Activities Index (FAI) [45] was used at inclusion and after the intervention to assess frequency of participation in everyday social and domestic activities over the last 3–6 months, thereby serving as a pre-stroke measure at inclusion for those participants with recent stroke. The scores range from 0 (inactive) to 45 (very active). For participants with recent stroke, a return to a pre-stroke score, as well as participants who had their stroke more than 6 months before inclusion, any improvement in score was considered a positive outcome.

### Secondary outcome measures

The Life Satisfaction Checklist (LiSat-11) [46] was used at 4 weeks after inclusion and after the intervention to assess the participants’ satisfaction with life in general. Scores were dichotomised into not satisfied (alternatives 1 to 4) and satisfied (alternatives 5 and 6) [47].

The Self-Efficacy Scale [48] was used at 4 weeks after inclusion and after the intervention to assess the participants’ confidence in their ability to perform 18 predetermined activities. The scores range from 1 (not confident at all) to 10 (completely confident) for each activity. A score of > 5 is considered to represent confidence in the ability to perform activities in daily life [48].

The Hospital Anxiety and Depression Scale (HAD) [49] was used at 4 weeks after inclusion and after the intervention to assess anxiety and depression. The HAD has two subscales, each ranging from 0 to 21. Scores were categorised as no anxiety and depression (0–7), mild (8–10) or moderate to severe anxiety and depression (10–21) [49].

The Fatigue Severity Scale (FSS) [50] was used at 4 weeks after inclusion and after the intervention to assess fatigue. The final score is the mean of the nine items graded between 1 (strongly disagree) and 7 (strongly agree). Scores were categorized as no fatigue (1–3) or fatigue (4–7) [51].

### Feasibility outcome measures

#### Fidelity

The team members were provided with a logbook template to take notes regarding their encounters with the participants in order to study the extent to which the delivery of the F@ce intervention followed the originally developed plan. The teams’ logbooks were collected at the end of the intervention. During the project, from the first workshop to final data collection (September 2017–February 2018), the second author was personally available to the teams each week (in conjunction with data collection) and via phone or email. Lunch meetings were also held with each of the teams around once a month in order to support the teams in their recruitment of participants and in conducting the intervention.

The second author also made field notes after each contact with the teams regarding their fidelity to F@ce.

#### Adherence

Data on goals and daily scorings of the participants with stroke were collected from the F@ce web platform in order to study how the participants adhered to the F@ce intervention. The maximum number of scorings for the participants was one scoring for each of the three goals a day, 7 days a week during the eight-week intervention.

#### Acceptability

Participants with stroke were asked to complete a survey containing open-ended questions about their experiences of F@ce concerning its potential benefits in rehabilitation and/or in everyday life, negative and positive aspects of using F@ce, as well as any technical difficulties/issues. The survey was distributed during the follow-up after the intervention.

#### Safety

Since falls are common after stroke the number of falls over the last 3 months were noted for each participant at inclusion and at follow-up after the intervention to monitor the safety of the intervention.

### Data analysis

Descriptive statistics were used to analyse and present the results regarding recruitment, the outcome measure used, adherence, acceptability, and safety. The team members’ fidelity to the F@ce intervention was analysed by comparing the descriptions of how the teams performed the intervention, as described in the teams’ logbook notes and the second author’s field notes, with the components of F@ce.

## Results

### Participants and recruitment

**Fig. 2:**
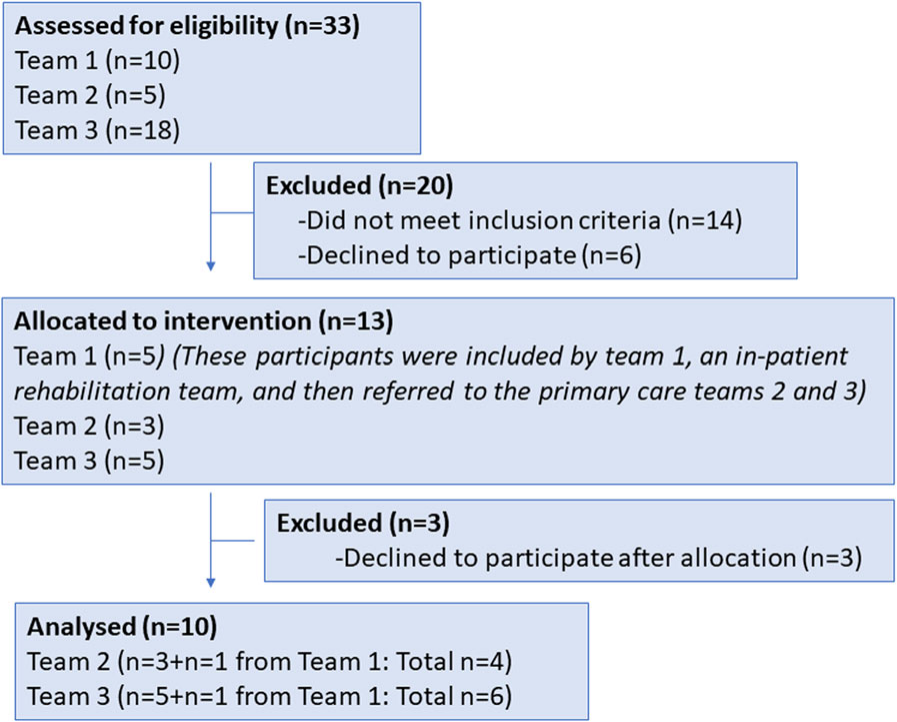
Flowchart of the recruitment of persons with stroke. The inclusion process of the participants in the study including assessed for eligibility, allocated to the intervention and the numbers of excluded

Two and a half weeks after the final workshop, the first eligible participant with stroke was identified and the recruitment started. Figure 1 shows the timeline of the study. From September–December 2017 a total of 10 participants were included to participate in the eight-week intervention, of which four participants started at an in-patent unit. Figure 2 illustrates the flowchart of the recruitment of participants with stroke. Out of 33 assessed for eligibility 13 were included i.e. the recruitment rate was 39%. Reasons for not meeting the inclusion criteria were: did not start or continue rehabilitation in any of the participating primary care units, severe fatigue, severe aphasia, foot fracture, and depression. Three participants withdrew participation after inclusion i.e. the dropout rate was 23%.

Mean age of the ten participants was 65 years (SD 12), five were men, four lived alone, and five had worked pre-stroke. All participants had suffered a mild stroke. Five had a recent stroke i.e. had suffered a stroke at ≤1 month before inclusion and five had suffered a chronic stroke i.e. from 10 to 32 months before inclusion. Three participants had a score on MoCA< 26 indicating cognitive impairment. However, one participant was unable to answer tall of the questions in MOCA due to aphasia and thus only scored 9. The participants’ current use of ICT was basic in one participant, moderate in three, and advanced in six participants.

### Primary outcome measures

#### COPM

All of the participants showed increased scores and a clinically meaningful improvement of ≥2 points was detected in the performance of four of the participants and in the satisfaction of six of the participants. Clinically meaningful improvements were found in both participants with recent stroke as well as participants with chronic stroke. (See Table 1).

**Table 1:** Participant goals and difference in the Canadian Occupational Performance Measure between inclusion and after the eight- week intervention

Pat	Goals	Goal 2	Goal 3	Difference in performance (mean)	Difference in satisfaction (mean)
1	Sing	Play the guitar	Give a lecture	1.7	6.7^a^
2	Write name	Brush teeth	Play the piano	1.2	0.7
3	Using the stairs	Walk outside	Water plants in garden	0.0	0.7
4	Ride the metro/ bus	Use a knife and fork	Tie shoelaces	7.4^a^	6.3^a^
5	Visit the gym	Ride the metro/bus	Return to work	5.4^a^	5.6^a^
6	Flip through pages in book	Reach for a glass	Open a mobile phone	0.7	0.3
7	Walk up and down a staircase	Visit and manage to use the toilet independently	Put on a t-shirt	2.3^a^	4.0^a^
8	Walk up and down a staircase	Reach for a glass	Make a sandwich	2.6^a^	2.6^a^
9	Walk using walking sticks	Take the initiative to carry out activities	Improve balance	1.9	4.3^a^
10	Improve handwriting	Use knife and fork	Pick up object with foot	0.3	0.3

^a^A clinically meaningful change in score of > 2

#### SIS 3.0

Clinically meaningful positive changes were shown in the domains of strength (*n* = 3), memory (*n* = 3), emotions (*n* = 3), participation (*n* = 3), recovery (*n* = 3), communication (*n* = 2), ADL/IADL (*n* = 2) and hand function (*n* = 1). Clinically meaningful negative changes were shown in the domains of hand function (*n* = 3), communication (*n* = 2), ADL/IADL (*n* = 2) and participation (*n* = 2) as well as mobility (*n* = 1), emotions (*n* = 1) and recovery (*n* = 1) (see Table 2).

**Table 2:** Participant outcomes according to the Stroke Impact Scale 3.0

Patient	1	2	3	4	5	6	7	8	9	10
Strength at 4 weeks	100	56	81	63	100	38	13	38	75	63
at follow up (0–100)	100	100^a^	94	69	100	56^a^	38^a^	31	88	50
Memory at 4 weeks	89	64	96	100	50	86	82	96	50	100
at follow up (0–100)	93	93^a^	100^a^	93	68^a^	86	68	96	43	100
Emotions at 4 weeks	58	67	100	94	58	78	58	89	58	64
at follow up (0–100)	92^a^	92^a^	56	86	70	69	44	94	47	88^a^
Communication at 4 weeks	93	64	100	100	79	93	89	57	71	100
at follow up (0–100)	93	93^a^	100	100	61	93	64	86^a^	68	100
ADL/IADL at 4 weeks	100	65	93	88	73	48	48	65	73	68
at follow up (0–100)	98	98^a^	65	96	96^a^	56	31	77	73	73
Mobility at 4 weeks	78	83	83	94	94	78	72	64	50	53
at follow up (0–100)	86	86	78	94	94	78	67	75	61	36
Hand function at 4 weeks	100	40	90	94	94	0	0	20	70	25
at follow up (0–100)	100	100^a^	85	94	94	0	0	5	10	10
Participation at 4 weeks	81	31	81	94	28	56	28	59	31	72
at follow up (0–100)	72	72^a^	63	88	53^a^	53	16	78^a^	41	56
Recovery at 4 weeks	78	30	70	70	50	60	35	40	75	60
at follow up (0–100)	70	45^a^	95^a^	75	80^a^	60	30	50	50	50

*ADL* Activities of Daily Living, *IADL* Instrumental activities of Daily Living

^a^A clinically significant improvement of > 15 points (or reaching the maximum score of 100)

#### FAI

Only one of the participants with recent stroke had returned to a pre-stroke level of participation. Four of the five participants with chronic stroke had improved their scores and one participant had a lower score compared to their score at inclusion (see Table 3).

**Table 3:** Participant outcomes according to FAI, HAD, LiSat-11, Self-efficacy scale, FSS

Patient	1	2	3	4	5	6	7	8	9	10
Months post stroke	< 1	< 1	1	< 1	< 1	10	19	22	24	32
FAI at inclusion	38^a^	33^a^	24^a^	34^a^	33^a^	17	5	12	18	13
at follow-up (0–45)	36	14	9	34	30	11	9	30	23	19
HAD-A at inclusion	3	8	1	11	2	13	3	1	10	1
at follow up (0–21)	2	6	4	3	4	2	10	1	9	0
HAD-D at inclusion	1	5	1	1	8	11	2	0	11	1
at follow-up (0–21)	2	4	3	0	10	6	16	3	9	0
LiSat-11 at 4 weeks	6	4	5	4	5	6	2	3	3	4
at follow up (1–6)	5	5	4	5	4	5	1	4	5	4
Self-efficacy scale at 4 weeks	7.7	6.2	8.1	9.8	8.4	7.1	5.0	7.4	6.3	7.8
at follow-up (1–10)	8.8	7.4	8.0	9.7	8.9	6.6	5.3	8.8	7.7	7.9
FSS at 4 weeks	5.1	6.7	2.6	1.9	6.3	4.6	5.8	4.3	6.8	1.3
at follow-up (1–7)	4.8	6.4	1.0	2.9	5.7	6.0	7.0	4.0	6.4	2.2

*FAI* Frenchay Activities Index, *HAD-A* Hospital Anxiety and Depression Scale – subscale anxiety, *HAD-D* Hospital Anxiety and Depression Scale – subscale depression, *LiSat-11* Life Satisfaction Checklist, *FSS* Fatigue severity scale

^a^Pre-stroke measure

### Secondary outcome measures

#### LiSat-11

Among participants with recent stroke, two reported a positive change from not satisfied to satisfied and two reported a negative change. Among participants with chronic stroke, only one reported a positive change.

#### Self-efficacy scale

All participants were confident in their ability to perform ADL at both 4 weeks and at follow-up (see Table 3).

#### HAD

Levels of depression and anxiety did not change in most patients. However, one participant with recent stroke had a positive change in score from moderate/severe anxiety to no anxiety. One participant with chronic stroke had a positive change from moderate/severe anxiety and depression to no anxiety and depression. Another participant with chronic stroke had a negative change from no anxiety and depression to mild anxiety and moderate/severe depression.

#### FSS

No changes regarding fatigue were shown.

### Feasibility of F@ce in terms of fidelity, adherence, acceptability and potential harm

#### Fidelity

According to the teams’ logbooks, overall fidelity to the components of F@ce was good i.e. the components of the interventions were followed by the participating team members and persons with stroke. In accordance with the first component of F@ce, the teams initially met the participants individually (i.e. face-to-face meetings) in their rooms at the in-patient unit, or in their homes, in the case of those participants who lived at home. Two team members were usually present. Team 3, which included participants with ongoing rehabilitation, made an additional home visit at inclusion in F@ce to start the intervention and set new goals. In many cases, participants stated that initial technical issues (lack of experience, lack of internet connection, etc.) took time and energy to solve and were frustrating for the teams and the participants with stroke.

The assessments were conducted according to the F@ce intervention using the mobile phone of the person with stroke to record a video of an activity as the basis for sharing and discussing the performance together with the participant. All participants’ progress was followed up by occupational therapists using the COPM after the end of the eight-week intervention. This was described as an extra effort for the therapists. Participants with recent stroke who had been admitted to the in-patient unit found it difficult to set realistic goals once they had returned home. For these participants, the goals or strategies were adjusted. The participants’ goals are presented in Table 1.

During the eight-week intervention the participants had individual or group sessions with the team (once/twice a week). Issues with goals, strategies or scoring were followed up at these meetings by the team instead of logging into the stroke rehabilitation platform in order to monitor the participants’ scorings. The follow-up meetings were described by the team members in the logbooks using unstructured field notes.

#### Adherence

According to data from the stroke rehabilitation platform, the participants responded to 44–100% of the text messages they received (mean 78%). According to the follow-up survey, five participants with stroke reported no technical difficulties, two reported some technical difficulties, two reported many technical difficulties and one reported that the technic did not work at all.

According to the researcher’s (second author) field notes, the teams contacted the researcher as soon as technical issues occurred in order to discuss potential solutions. One participant was unable to use their mobile phone (or tablet) to rate the goals and was therefore asked to write their ratings on paper after a discussion with the team members. Another participant who lived in a rural area had difficulties with the internet connection and therefore wrote their ratings on paper for 79% of the time. Additionally, two participants experienced initial problems using the stroke rehabilitation platform before they learnt how to register their ratings; they would write their ratings on paper when necessary.

#### Acceptability

According to the follow-up survey, all of the participants (100%) stated that using F@ce had been positive and described the overall experience as interesting (*n* = 6), enjoyable (*n* = 2), supportive (*n* = 1) or a reminder (*n* = 1). All of the participants stated that F@ce had supported their rehabilitation to a very high extent (*n* = 5), a high extent (*n* = 2) or to some extent (*n* = 3). The participants also stated that F@ce had supported their everyday lives to a very high extent (*n* = 2), a high extent (*n* = 3), to some extent (*n* = 4) or not at all (*n* = 1).

Five participants stated that visualizing their goals, being reminded and aware of their abilities and how to perform their activities were perceived as a benefit of F@ce. Three participants stated that exercising more and “having to perform their activities was positive”. One of the participants stated that everything associated with F@ce was positive and another participant stated that it gave them an opportunity to utilise once memory function and the mobile phone.

Regarding the negative aspects of F@ce, two of the participants wished that they had been able to adjust their goals along the way. One participant referred to the technical issues and three participants stated that being “forced” to do the activities and feeling guilty when they did not do the activities were negative aspects. Some of participants would recommend F@ce to someone else without a doubt (*n* = 5) or would be likely (*n* = 5) to recommend F@ce to someone else.

Among the participants with recent stroke, one reported falling once before leaving the hospital and another reported falling four times after returning home. Among the participants with chronic stroke, one reported falling two times before the intervention and three times during the intervention. Another participant with chronic stroke reported falling two times before the intervention and not falling at all during the intervention. No additional harm or safety issues were reported in the logbooks or researcher’s field notes.

## Discussion

The results of this feasibility study indicate that the F@ce intervention was feasible to use within both in-patient and primary care rehabilitation after stroke. The outcome measures that were used were feasible and took approximately 20–40 min to complete. Even though this study was not designed to evaluate the effects of F@ce as such, clinically significant improvements in the COPM and in the SIS were found in several of the participants after only 4 weeks, which is seen as promising. Overall, the participating teams and the persons with stroke were satisfied with F@ce, and adherence and acceptability were high. The fidelity of the teams to the intervention requires some improvement, for example, more time for workshop planning and preparation and better procedures for team members for following-up the intervention.

One of the main findings was that persons with stroke appreciated and adhered to F@ce to a large extent. In particular, they stated that the goals they had formulated, and the daily alerts were beneficial for their recovery. The results of the COPM also showed that the participants’ perceived performance and satisfaction increased. Thus, also using the COPM [40] as part of the intervention appeared to be appropriate and the therapists reported no difficulties in using the measurement. A culturally and contextually adapted version of the F@ce intervention showed that the COPM was usable and also showed significant results when evaluated in Uganda [12]. In this study, the COPM was performed by occupational therapists only, possibly because they were more used to using the instrument and because it was originally developed by occupational therapists. Other team members may therefore not have been accustomed to using the instrument. However, the COPM has previously been used in a team-based intervention and has been shown to improve person-centredness and participation in goal setting [52]. It could be that more introduction and guidance is required in using the instrument since it is considered appropriate for physiotherapists to use the instrument [53]. Following a stroke and/or other brain injuries, self-awareness could be an issue that could make goal setting difficult [47]. Nevertheless, it has been shown that the COPM can be used to set goals despite self-awareness issues, although support from significant others or a therapist may be necessary [54].

The F@ce intervention was developed to meet the current and future needs for rehabilitation of stroke patients. In this century, progress in the field of medical research has been greater than ever before in areas such as the development of new treatments and providing high-quality care [55]. Swedish health care is ranked amongst the best in the world in treating cancer, acute illness and vaccinations [51]. However, when it comes to caring for people with long-term illnesses, safe patient care and patient satisfaction, Swedish health care is ranked amongst the bottom third of over 40 countries worldwide [51]. The Swedish Health and Social Care Inspectorate (IVO) has reported that there are flaws in person-centredness and in the coordination of care in the Swedish healthcare system [56]. The IVO further reports the need to develop digital tools that are simple and usable for communication and follow-up, as well as to create a model for inter-professional teamwork [56]. The development of F@ce has taken such needs into account when creating a model for team-based rehabilitation with the support of ICT in order to enhance communication and enable follow-up from a distance. According to the teams’ logbooks, the overall fidelity to the components of F@ce was good. Also, even though the team had no daily contact and communication with the participants, they appeared to be motivated by the daily alerts and the rating system. Some of the participants reported that the goals needed to be adjusted more frequently, which could indicate that the professionals required further support in how to monitor and follow up the patients’ ratings.

Nevertheless, the results indicate that the most beneficial aspects of F@ce was the person-centred goal-setting process and SMS alerts. The findings show that the participants set their goals based on activities they needed and/or wanted to perform in their everyday lives. This is in line with the results of the F@ce study in Uganda in which all participants were positive about the reminder system and felt that it helped them regain their abilities and that the follow-up system was beneficial to their rehabilitation process [16]. According to the national stroke guidelines [31] and rehabilitation research, setting goals is an essential part of the rehabilitation process [57, 58]. This study shows that team members are important for providing support and guiding goal setting, but also for adjusting the goals during the rehabilitation process.

The time spent on preparing and training the teams to use F@ce was restricted to a two-hour workshop once a week for 3 weeks. This was less time for preparation compared to other studies performed within the research group. For example, in the CADL study, there were five full days of preparation over one month [28] and in Uganda the participating therapists took part in a series of workshops over eight half days [12]. In the evaluations of the implementation of the CADL intervention, the collaborative relationship between the occupational therapists and the researchers was described as a relationship that enabled the fusion of scientific knowledge and practice [27]. Thus, it is important for researchers to spend sufficient time building a relationship with professionals and sharing knowledge and experiences using a “healthcare professional-centredness” perspective. Successful implementation could also depend on personal factors such as if the professionals are motivated and have sufficient knowledge of the underlying theory and the implementation process [59]. Organisational factors such as having the necessary resources and support from management, as was the case in this study, have also been shown to be important [59]. Thus, flexible and supportive collaboration throughout the implementation process is important, especially when something unexpected occurs. During the implementation of F@ce, the second researcher was present at the units each week while collecting data and was therefore able to maintain a relationship with the teams and provide support. However, in future testing and implementation of F@ce, it would be beneficial to prolong the workshops and have an even closer collaboration between professionals and researchers in order for the teams to have time to implement new knowledge in relation to the intervention.

The stroke rehabilitation web platform was developed to provide an opportunity for the teams to collaborate with the participants by sharing their daily ratings, enabling follow-up when necessary. However, this platform was not used to the extent expected. Team members stated that they lacked the time or that using the web platform had not yet been incorporated into their routines. Instead, they usually followed-up the participants’ ratings and progress in face-to-face meetings. Some of the participants with stroke stated that they wanted better follow-ups and adjustments of their goals. These results are in line with previous research that highlights the challenges of working with person-centredness within a team, including communication and collaboration with a person and within a team as key elements of goal setting and rehabilitation planning [60].

A limitation of this study could be that several outcome measures, for example, SIS, were used at four and 8 weeks after inclusion, i.e. only 4 weeks apart. It would have been preferable if the inclusion and follow-up could have been further apart to identify plausible changes over time. Furthermore, the F@ce intervention needs to be evaluated through qualitative interviews with users, team members, patients, and their significant others in order to evaluate their experience of participating in the intervention. Health economic evaluations should be performed to analyse the cost of the intervention in terms of purchasing hardware and software. Another limitation is the lack of control group and the small sample size. A larger sample size could have provided greater precision of scores for the outcome measures. However there is no definitive sample size recommended for feasibility studies, rather a range from 10 to 50 participants or more [61]. A large scale RCT study needs to be performed to evaluate the effects of F@ce. The recruitment rate of 39% indicates a need for clearer inclusion criteria and a close communication with the recruiting team member during the recruitment process. However, only three participants dropped out after inclusion which is a promising result of the intervention. It should be noted that all participants had had a mild stroke. Nevertheless, a strength of the study is that F@ce was evaluated and found to be beneficial for participants with a recent stroke as well as for participants who had a stroke several years ago.

Lastly, in this study, ICT was used as a tool throughout the rehabilitation process to enable sharing and transparency between the rehabilitation team and participants with stroke by providing them with alerts and feedback. This is in line with the results of a recent scoping review which shows that ICT can be used as an alternative to face-to-face interventions in order to improve participation in daily life after stroke [3]. Nevertheless, it is important to consider which individuals to target so that nobody is excluded from rehabilitation when using ICT. For example, older people might prefer face-to-face interactions or phone calls in their contact with healthcare professionals and for such people non-digital alternatives must be available [62]. Thus, it is important to provide support for people who are inexperienced in the use of ICT or who have cognitive or physical impairments that might hinder their use of ICT. However, since the use of ICT support in health care and rehabilitation will probably be necessary in the future, the development and evaluation of the F@ce intervention have contributed to such a digital development process.

## Conclusion

The F@ce interventions appeared to remind and inspire the participants to perform activities and to improve their participation in daily activities after stroke. However, the teams must identify routines for follow-up in order to ensure that they provide appropriate support.

Using the COPM seems to be suitable for evaluation of this type of interventions. Additionally, the results of this study found that several of the patients improved their self-perceived performance and satisfaction of the activities according to the COPM over 8 weeks.

## Supplementary information

**Additional file 1 Supplementary Figure 3**. Components of the F@ce intervention modelling from CADL,^1,2,3^. Two general strategies are combined and should be used by the teams (i.e. during the entire intervention process) in order to enable change: 1) using the client’s lived experience as a point of departure and 2) enabling significant experience to be gained from performing valued daily activities.

## Data Availability

The datasets supporting the conclusions of this article are available at the Division of Occupational therapy, Department of Neurobiology Care Sciences and Society, Karolinska Institutet, Stockholm, Sweden. E-mail: susanne.guidetti@ki.se.

## Abbreviations

ADL: Activities in Daily Living
CADL: Client-centred ADL intervention
COPM: Occupational Performance Measure
ICT: Information and Communication Technology
FAI: Frenchay Activities Index
FSS: Fatigue Severity Scale
HAD: Hospital Anxiety and Depression Scale
LiSat-11: Life Satisfaction Checklist
MoCA: Montreal Cognitive Assessment
MRC: Medical Research Council
RCT: Randomized Controlled Trial
SIS: Stroke Impact Scale
