# Supplementary material for: F@ce: a team-based, person-centred intervention for rehabilitation after stroke supported by information and communication technology – a feasibility study
Source: BMC Neurol. 2020 Oct 23;20:387. doi: 10.1186/s12883-020-01968-x (PMC7583214; doi:10.1186/s12883-020-01968-x)
Supplement: Supplementary file 1 — Additional file 1 Supplementary Figure 3. Components of the F@ce intervention modelling from CADL,1,2,3. Two general strategies are combined and should be used by the teams (i.e. during the entire intervention process) in order to enable change: 1) using the client’s lived experience as a point of departure and 2) enabling significant experience to be gained from performing valued daily activities. [file 12883_2020_1968_MOESM1_ESM.docx]

**Supplementary Figure 3. Components of the F@ce intervention modelling from CADL ,**^1,2,3^

Two general strategies are combined and should be used by the teams (i.e. during the entire intervention process) in order to enable change: 1) using the client’s lived experience as a point of departure and 2) enabling significant experience to be gained from performing valued daily activities.

| **F@CE^TM^** | **Intervention** | **Basic principles** |
| --- | --- | --- |
| **F**  **Face-to-face meeting** | The first meeting: build a relationship with the patient. Provide relevant information including contact information. | Create a therapeutic relationship^4^ with the patient and adopt person-centred approach^4-8^. |
|  | Allow the patient to describe their abilities, roles and habits (past and present). | Base the rehabilitation on the patient’s performance of daily activities ^4, 8 ,9, 10, 11,^ their unique life experiences^12^ and narrative ^4, 8, 11, 13^. |
|  | Make contact with family/significant others, provide relevant information, including contact information. | Involve significant others by providing information and support ^14-17^. |
| **A**  **Assessments** | Allow the patient to describe their performance of daily activities. Record an activity on video and let the patient rate their performance. | Sharing is important in a person-centred approach ^18^. Shared assessment, to have common ground for planning the rehabilitation ^4^. |
|  | Use the COPM as a basis to create goals. | Use the Canadian Occupational Performance Measure (COPM) ^10^, a person-centred outcome measure, for setting goals for each patient. |
| **C**  **Collaboration** | Set three goals and create a clear plan with strategies to work on both individually and together with the team. | Be transparent in communication and information in order to achieve a person-centred rehabilitation ^19^. Enable change through setting goals and formulate strategies on the F@ce web platform. |
|  | The patient receives daily alerts through the F@CE web platform and rates their performance each day. The teams are able to keep track of each patient’s process through F@CE and offer support to patients and significant others when needed. | Ensure that the patient is actively involved in goal setting and planning rehabilitation ^1, 2,^ ^4, 8, 9, 10^.  Monitor the patient’s progress through the ratings ^20^. |
| **E**  **Evaluation** | Evaluation through the COPM at the end of the eight-week intervention. | Use the COPM at follow-up to evaluate. A difference of two or more between first assessment and follow-up is considered to be clinically significant ^21^. |
|  | Plan continued rehabilitation by revising the goals or by referring to other units or other professionals. |  |

References to supplementary Figure 3

1. Bertilsson AS, Ranner M, von Koch L, Eriksson G, Johansson U, Ytterberg C, et al. A client-centred ADL intervention: three-month follow-up of a randomized controlled trial. *Scand. J. Occup. Ther., 2014, Vol21(5), p377-391*. 2014; 21: 377-91.
2. Guidetti S, Ranner M, Tham K, Andersson M, Ytterberg C, Von Koch L. A client-centred activities of daily living intervention for persons with stroke: One-year follow-up of a randomized controlled trial. *J Rehabil Med.2015; 47: 605*
3. Kamwesiga T J, Eriksson G, Tham K, Fors U, Ndiwalana A, von Koch L, et al. A feasibility study of a mobile phone supported family-centred ADL intervention, F@ce, after stroke in Uganda. *Globalization and health*. *2018; 14: 82*
4. Guidetti S,Tham K. Therapeutic strategies used by occupational therapists in self-care training: a qualitative study. *Occup Ther Int. 2002; 9: 257-76*
5. National Board of Health and Wellfare. Swedish national guidelines for stroke care 2017. Stockholm, Sweden: National Board of helath and wellfare, 2017.
6. Rogers CR. *Client-centered therapy : its current practice, implications, and theory*. Boston: Houghton Mifflin, 1951.
7. Rogers CR. *Client Centred Therapy (New Ed) [Elektronisk resurs]*. New York: Constable & Robinson, 2012
8. Ekman I, Swedberg K, Taft C, Lindseth A, Norberg A, Brink E, Carlsson J, et al. Person- Centered Care — Ready for Prime Time. *European J Cardiovasc Nurs.* *2011; 10: 248-51*
9. Taylor RR. *Kielhofner's model of human occupation: theory and application*. Philadelphia: Wolters Kluwer, 2017.
10. Townsend EA, Polatajko HJ. *Enabling occupation II : advancing an occupational therapy vision for health, well-being & justice through occupation*. Ottawa: CAOT Publications ACE, 2007.
11. Guidetti S, Asaba E, Tham K. The lived experience of recapturing self-care. Am J Occup Ther.2007; 61: 303–310
12. Merleau-Ponty M. *Phenomenology of Perception*. London: London : Routledge, 1989.
13. Guidetti S, Asaba E,Tham K. Meaning of context in recapturing self-care after stroke or spinal cord injury. *Am J Occup Ther.2009; 63: 323*
14. National board of health and welfare. Lifesituation two years after stroke- A follow up of people after stroke and their significant others. National board of health and welfare, 2004.
15. Bergstrom AL, von Koch L, Andersson M, Tham K, Eriksson G. Participation in everyday life and life satisfaction in persons with stroke and their caregivers 3-6 months after onset. *J Rehabil Med*. *2015; 47: 508-15*
16. Bertilsson AS, Eriksson G, Ekstam L, Tham K, Andersson M, von Koch L, et al. A cluster randomized controlled trial of a client-centred, activities of daily living intervention for people with stroke: one year follow-up of caregivers. *Clin Rehabil*. *2016; 30: 765-75*
17. Bertilsson A-S, Von Koch L, Tham K, Johansson U. Client-centred ADL intervention after stroke: Significant others' experiences. *Scand. J. Occup. Ther.* *2015; 22: 377-86*
18. Ranner M, Von Koch L, Guidetti S, Tham K. Client-centred ADL intervention after stroke: Occupational therapists’ experiences. *Scand. J. Occup. Ther.* *2016; 23: 81-90*
19. Ranner M, Guidetti S, Von Koch L, Tham K. Experiences of participating in a client-centred ADL intervention after stroke. *Disabil Rehabil.* 2018: 1.
20. Fors U, Kamwesiga JT, Eriksson GM, von Koch L, Guidetti S. User evaluation of a novel SMS-based reminder system for supporting post-stroke rehabilitation. *BMC Med Inform Decis Mak.*2*019; 19: 122*
21. Law M. *Canadian occupational performance measure : svensk version*. Stockholm: Förbundet Sveriges arbetsterapeuter, 2006.
